# Prevention and Control of *Legionella* and *Pseudomonas* spp. Colonization in Dental Units

**DOI:** 10.3390/pathogens9040305

**Published:** 2020-04-21

**Authors:** Benedetta Tuvo, Michele Totaro, Maria Luisa Cristina, Anna Maria Spagnolo, David Di Cave, Sara Profeti, Angelo Baggiani, Gaetano Privitera, Beatrice Casini

**Affiliations:** 1 Department of Translational Research, N.T.M.S., University of Pisa, 56123 Pisa, Italy; tuvobenedetta@hotmail.it (B.T.); micheleto@hotmail.it (M.T.); profeti.sara@gmail.com (S.P.); angelo.baggiani@med.unipi.it (A.B.); gaetano.privitera@med.unipi.it (G.P.); 2 Department of Health Sciences, University of Genova, Via Pastore 1, 16132 Genova, Italy; cristinamle@unige.it (M.L.C.); am.spagnolo@unige.it (A.M.S.); 3 Department of Clinical Sciences and Translational Medicine, University of Rome “Tor Vergata”, 00133 Rome, Italy; dicave@uniroma2.it

**Keywords:** Dental unit waterlines, *Legionella* spp., risk management, disinfection

## Abstract

**Introduction:** Dental Unit Waterlines (DUWLs) have shown to be a source of *Legionella* infection. We report the experience of different dental healthcare settings where a risk management plan was implemented. **Materials and methods:** In a Hospital Odontostomatology Clinic (HOC) and three Private Dental Clinics (PDCs) housing 13 and six dental units (DUs), respectively, an assessment checklist was applied to evaluate staff compliance with guideline recommendations. DUWLs microbial parameters were investigated before and after the application of corrective actions. **Results:** In the HOC a poor adherence to good practices was demonstrated, whereas protocols were carefully applied in PDCs. *L. pneumophila* sg 2–15 was isolated in 31% (4/13) and 33% (2/6) of DUs in HOC and PDCs, respectively, mainly from handpieces (32%, 6/19) with counts >10^2^ colony-forming units per milliliter (CFU/L), often associated with *P. aeruginosa* (68%, 13/19). The shock disinfection with 3% v/v hydrogen peroxide (HP) showed a limited effect, with a recolonization period of about 4 weeks. *Legionella* was eradicated only after 6% v/v HP shock disinfection and filters-installation, whilst *P. aeruginosa* after the third shock disinfection with a solution of 4% v/v HP and biodegradable surfactants. **Conclusions:** Our data demonstrate the presence and persistence of microbial contamination within the DUWLs, which required strict adherence to control measures and the choice of effective disinfectants.

## 1. Introduction

Several studies have demonstrated that water output from Dental Unit Waterlines (DUWLs) is often contaminated with high densities of microorganisms [1,2], ranging from 10^2^ to 10^6^ colony-forming units per milliliter (CFU/mL) [3,4,5,6,7,8,9,10]. Bacterial biofilm may be present on the inner surfaces of DUWLs due to contaminations coming from the proximal or distal portion of the circuit. In particular, the presence of small narrow-bore hydrophobic polymeric plastic tubing that facilitate the microorganisms’ adhesion (2 mm diameter), electrical components that can heat the water (20–25 °C) and the discontinuous and low water flow are all factors that contribute to microbial growth and biofilm formation. The biofilm remains fixed to the tubing wall, but microbes keep spreading from the biofilm into the water as it flows through. For this reason, high microbial levels have been found in output water from handpieces and air/water syringes [11,12]. DUWLs are equipped with a dual water supply system that permits the system to be supplied with only municipal water or sterile water or with both types. Water supply is usually provided by public utilities and its quality must comply with the parameters required by law [13].

The suck-back of biological fluids from the oral cavities of patients (back-contamination) was also reported as important cause of DUWLs contamination [14,15]. 

*Pseudomonas* spp. are the prevalent bacteria found in DUWLs but a high incidence of *Legionella* was also reported, widely varying from 0% to 68% [16] (including *Legionella pneumophila* serogroup 1) [17]. DUWLs may also be important replication sites for free-living amoebae and protozoa that enable the maintenance of pathogenic intracellular bacteria, increasing their resistance to disinfectants [18]. There is evidence that amoebae huddle around microbial biofilm, and that their concentration is up to 300 times higher in DUWLs’ output water than in tap water from the same source [15,16,17]. The microbial adhesion and biofilm on DUWLs surfaces remain very difficult to eradicate.

The Centers for Disease Control and Prevention (CDC) recommends that the maximum level of non-coliform bacteria emitted from dental handpieces and air/water syringes should be equal or less than 500 CFU/mL [19]. The Italian legislation on drinking water does not establish a limit for heterotrophic bacteria count; it requires this value not to undergo variations. The water in the operating theatre of healthcare facilities must comply with the target value established by the Italian Workers Compensation Authority guideline, 100 CFU/mL at 22 °C and 10 CFU/mL at 37 °C, respectively. In 2007, the French “*Ministère de la Santé et des Solidarités*” proposed the same target for drinking water. For this reason, we took it as a reference in our study [20,21].

Because of this contamination, dental units are recognized as a potential source of infection for human health, especially threatening dental staff and patients, who are regularly exposed to water and water-aerosol emitted by dental unit handpieces. A study reported the case of a patient who died after being infected with *L. pneumophila* serogroup 1 during a dental practice in Italy [22]. By different typing methods, it was demonstrated that the source of the *Legionella* infection was the DUWL. *L. pneumophila* sg 1 subgroup Benidorm ST593 was isolated in each sample collected from the cold tap water, tap of the DUWL, high-speedturbine and patient’s bronchialaspirate. A study of Schönning et al. reported the case of a man diagnosed with leukaemia, who underwent a dental check-up and a high-dose chemotherapy and developed Legionnaires’ disease in the following days. The analysis of the environmental samples, from the cup filler resulted to be positive for the same *L. pneumophila* sg 1 specimen detected in the patient’s sputum collected through bronchoscopy [23]. 

The evidence linking exposure to *Pseudomonas aeruginosa* contaminated DUWL during dental treatment and subsequent infection is limited [24] and is based on the results from a single observational study reported in two cancer patients by Martin M.V. et al. [25]. 

Free-living amoebae were frequently isolated from DUWLs and *Vermamoeba* species were isolated from the throats of humans as long ago as 1967 [26] but it is unknown whether they are associated with a risk in the dental setting, through contaminated aerosols or droplets [27].

In literature there is a single documented fatal case of Legionnaires’ disease regarding an American dentist. The infection was attributed to exposure to DUWL aerosols [28]. *L. pneumophilia* and *L. longbeachae* were detected in the dentist’s lung tissue and in the DUWLs; however, the dentist’s domestic water supplies also had very low levels of *Legionella* spp. Here, the evidence was not conclusive. 

A meta-analysis conducted by Petti S et al. demonstrated that there was limited evidence of occupational risk for *Legionella* infection to dental healthcare workers and Legionnaires’ disease outbreaks in dental healthcare settings were never reported despite billions of treatments provided each year [29].

To date, there are no known cases of Pontiac fever in patients, resulting from visits to, or treatment in, a dental clinic. This would suggest that the risk to patients posed by *Legionella* spp. from DUWLs is low; however, the risk is not absent [30].

The aim of this study was to report the experience of different dental healthcare settings where a risk management plan was implemented and an integrated filtration-disinfection strategy was applied to reduce the DUWLs microbial contamination. 

## 2. Results

### 2.1. Risk Management

Using the checklist allowed us to verify that a water safety plan was in place in the hospital and a maintenance and control program was constantly applied to the water of the buildings and to the aeration systems. This should ensure a good quality of municipal water that can feed the DUWL when the switching is applied if the sterile water in the bottle runs out. 

Despite the training activity for the correct adoption of the DU management procedure (using only sterile reverse osmosis water, flushing between patients, self-contained water bottles disinfection, etc.), a low adherence to good practices was found in the hospital.

On the contrary, a good compliance with manufacturer’s instructions for DU management and use of biocides was observed in the private dental clinics, where the staff was not informed/formed neither on water risk assessment and management nor on good practices guidance.

### 2.2. Tap Water Results

The microbiological quality of tap water varied between hospitals and smaller premises. *Legionella* spp. was not detected in three Private Dental Clinic (PDC) tap water samples (0/48), whilst in Hospital Odontostomatology Clinics (HOC), housing 13 dental units, *Legionella* spp. was repeatedly isolated in all tap water samples (30/104, 28.8%). The strains were identified as *L. pneumophila* sg 1 and sg 2–15, with a geometric mean of 4.91 ± 0.69 Log CFU/L and 4.38 ± 0.72 Log CFU/L, respectively. *Pseudomonas aeruginosa* and coliform bacteria were not isolated despite the fact that the total microbial counts at 22 °C and 37 °C were higher than the values recommended by Italian regulations (geometric mean of 2.79 ± 0.40 Log CFU/ml and 1.88 ± 0.41 Log CFU/ml, respectively). Free-living amoebae were recovered in 23% (3/13) of hospital DUs, but never in DUs housed in the PDCs. Among all cells microscopically positive to the culture examination, all PCR positive isolates belonged to *Vermoamoeba vermiformis* (identity of 99%). Despite the continuous application of the water safety plan, the microbiological quality of the municipal water remained low in the hospital and an inadequate concentration of residual chlorine-dioxide was detected (0.07 ± 0.14 mg/L). The mean temperature in cold water was 22.4 ± 1.6 °C, and the values were demonstrated to be related to *Legionella* concentration (R^2^ = 0.51). 

### 2.3. Dental Unit Results before Shock Disinfection

During the first sampling, water samples collected from hospital DUWLs showed a high prevalence of *Legionella*, which was detected in 31% (4/13) of dental units with a geometric mean 3.99 ± 0.61 Log CFU/L, often at different sites on the device (Table 1). *Legionella* was isolated from inlets (3/4), spittoons (2/4) and from handpieces (4/4). Positive isolates were identified as *Legionella pneumophila* serogroup 2–15. 

In PDCs, *Legionella pneumophila* serogroup 2–15 was isolated in 33% (2/6) of dental units during the first sampling, with a geometric mean of 4.15 ± 0.13Log CFU/L. *Legionella* was isolated from spittoons (2/2) and from handpieces (2/2).

**Figure 1:**
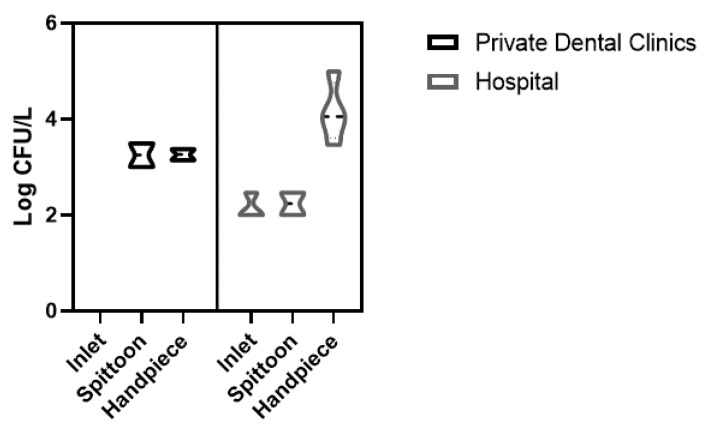
Violin plot of *Legionella pneumophila* sg 2–15 (Log colony-forming units per milliliter (CFU/L)) detected from each sampling site of the dental units before disinfection.

All *Legionella* data collected before dental unit waterlines’ disinfection are shown in Figure 1.

No coliform growth was detected in water samples. *P. aeruginosa* was isolated in high prevalence from handpieces and spittoons in 68% (13/19) of dental units. *P. aeruginosa* was detected in 33% (2/6) of collected samples, during the first sampling, from the spittoons of private clinics’ dental units and in 85% (11/13) of dental units housed in the hospital clinic. *P. aeruginosa* was always associated with the presence of *Legionella*. In almost all water samples, the total microbial counts at 22 °C and 37 °C was ≥ 100 and 10 CFU/mL, respectively.

*Brevundimonas vesicularis* was identified in two/six of the dental units housed in private clinics. Colonies were selected from Cetrimide Agar medium. All the percentages of positive dental units to waterborne pathogens are shown in Table 1.

Free-living protozoa (FLA) were detected in 46% (6/13) of HOC dental units. Among all FLA microscopically positive to the culture examination, one isolate showed band with approximate sizes of *Valkampfia.* All PCR positive isolates showed bands with approximate sizes of 800 bp (expected for *Vermoamoeba vermiformis*). The analyses unambiguously identified all samples as *Vermoamoeba vermiformis*, the sequences showing the highest identity (99%) with those accessible in GenBank. No water sample analyzed in this study was characterized for the presence of other FLA species, including *Naegleria* spp. Concerning *V. vermiformis* sequences, a phylogenetic analysis was also performed.

### 2.4. Dental Unit Results after Shock Disinfection and Water Filtration

In only one Private Dental Clinic was shock disinfection with hydrogen peroxide (HP) 3% v/v performed for pathogens (*P. aeruginosa* and *Legionella* spp.) in the DUWL, and filters were simultaneously installed at the inlet of each dental unit. Disinfection and filter installation showed a good efficacy on *Legionella* spp., undetected after the treatment, as well as on *P. aeruginosa*. No other treatment was needed. 

After 30 days from shock disinfection treatments with HP 3% v/v applied in 12 dental units in the HOC, total microbial counts at 22 °C and 37 °C resulted in the values being higher than the values recommended by Italian regulation (<100 CFU/mL and <10 CFU/mL, respectively) in almost all samples (67% and 92% respectively). *Legionella* spp. was detected in only one dental unit (8%), whereas *P. aeruginosa* was isolated in 83% of dental units (10/12), from handpieces and spittoons. The shock disinfection with HP 3% v/v showed a limited effect, with a recolonization period of about 4 weeks.

*Legionella* was eradicated after a shock disinfection with HP 6% v/v, applied after the installation of 31 days membrane filters at the inlet of each dental unit. The point-of-use water filtration showed good efficacy in containing the entrance of *Legionella*, while HP disinfection had a good performance in controlling the growth of pathogens. After 30 days from disinfection with HP 6% v/v, *P. aeruginosa* was still found in 100% of DUWLs’ samples (10/10), showing the higher persistence of this bacteria even after shock disinfection treatment. *P. aeruginosa* was not detected only after the third shock disinfection applied with a solution containing HP 4% v/v and surfactants.

All the results of the microbiological analysis performed on water samples collected from dental units housed in a Private Dental Clinic and in the Hospital Dental Clinic before shock disinfection are shown in Table 2.

## 3. Discussion

The dental unit consists of a complex water pipeline network connected to multiple pieces of equipment. Such an environment implicates a high risk of microbial contamination and transmission, especially through the contamination of water and bioaerosol by dental instruments, which are placed very close to patients and medical staff during dental treatments [31].

Even though the evidence associating DUWLs with risks for patients and staff is contradictory [32,33,34,35], exposing patients or dental healthcare personnel to water of uncertain microbiological quality is inconsistent with general accepted infection control principles [19]. Since patients and dental staff are regularly exposed to water and aerosols generated from the dental units, the microbial quality of the water in the DUWLs is extremely important. It is not acceptable that a DUWL should not meet the drinking water standards (<100 CFU/mL at 22 °C and <10 CFU/mL at 36 °C) [20,21].

To reduce the risk coming from contaminated DUWLs, it is necessary that dental healthcare workers routinely apply suggested infection prevention strategies [13]. The Italian guidelines suggest many approaches to reduce the microbial contamination and/or biofilm formation, including both non-chemical (using the anti-stagnation device, flushing, supplying the circuit with sterile solutions, antimicrobial filter installation, etc.) and chemical methods, which provide for the use of disinfectants continuously or in periodic shock treatment, following the manufacturer’s instructions [36]. Not an available single method or device will eradicate the bio-contamination of DUWLs or exclude the risk of cross-infection. To reduce contamination risks, a combination of methods is desirable [36]. Where delivered water quality is in doubt, dental practice should consider adopting continuous dosing systems or shock disinfection, if permitted by the manufacturer’s instructions [19]. Output water from DUs continuously treated with disinfection products is more compliant with the recommended standards (heterotrophic bacteria load) and it is reported to be remarkable in preventing the contamination by *Legionella* and *P. aeruginosa* [8,36,37]. The efficacy of the adopted measures depended on the strict adherence to the planned protocols [36].

The level of *Legionella* contamination in DUs is not established in the Italian Legislation. Nevertheless, all control actions to reduce the risk of contamination, biofilm formation in DUWLs and a risk assessment based on patients and clinical practices are required [13].

Besides the technical-practical measures and disinfection protocols, an integrated approach for microbial risk management in a dental health care setting should also include regular microbiological monitoring. Environmental surveillance for *Legionella* is useful not only to assess the efficacy of preventive measures but also as a guide for the choice of corrective strategies, under the principles of the internal control plan [38].

Our study confirms the literature evidences with the finding of *Legionella* or *P. aeruginosa* contamination in a large part of our samples before disinfection: 32% (6/19) and 68% (13/19) respectively. *P. aeruginosa* inside DUWLs may be related to the water quality or to the retro-contamination at the outlet of dental units. The competitive advantage of *P. aeruginosa* in the colonization of water lines is because of its ability to inhibit the growth of other bacteria by producing bacteriocins [39,40].

Frequent switchover from reverse osmotic to drinking water resulting from the intensive dental care activity in the hospital clinic, may have caused the higher *Legionella* spp. and *P. aeruginosa* isolation observed in the hospital DUWLs compared to private dental clinics. In large hospital buildings, drinking water quality may affect the DUWLs microbial contamination (more complex plumbing systems, large water storage tanks, multiple dead legs, cold water over 20 °C, etc.). 

As reported by Lizzaro et al., the possibility of switching between two different water flows (municipal water and sterile water) reduces the risk of circuit contamination, but a mixed water supply is not recorded in water safety plans [31].

In the Hospital Dental Clinic (HDC), a Water Safety Plan (WSP), a maintenance and a control program were constantly applied to the building’s water and aeration systems but there was a low adherence to good practices in DU management, despite the training activity of the dental healthcare staff on the correct adoption of the hospital procedure (exclusive use of sterile reverse osmosis water, flushing between patients, self-contained water bottles disinfection, etc.). A low adherence to the best practices guidance may also contribute to biofilm proliferation. On the contrary, a good compliance with manufacturer’s instructions for DU management and the use of biocides was observed in PDCs, although the staff was not informed on either on water risk assessment and management or on good practices guidance. The medical staff’s and manufacturers’ poor knowledge about water quality and the role of biofilm formation was suggested as one of the main problems related to dental unit contamination [31].

In only one Private Dental Clinic was shock disinfection with hydrogen peroxide 3% v/v and filters simultaneously installed at the inlet of each dental unit proven effective in eliminating pathogens, although the bacterial loads remained too high. These results were in line with the study conducted by Ditommaso et al., that showed an increase to unacceptable levels of bacterial loads in the DUWL of a dental chair treated with HP 3%, getting a significant reduction of counts only after 9 months of treatment [41].

On the other hand, in the hospital dental clinic, the first shock disinfection with HP 3% v/v applied in 12 dental units reduced positive samples for *Legionella* to 8% (1/12 DUs), and after the second treatment with HP 6% v/v and filters installation *Legionella* was removed. Conversely, after HP 6% v/v disinfection, *P. aeruginosa* proved to be more resistant and was detected again after two cycles of treatment, giving no evidence of substantially decreasing (100%, 10/10). Only after a third cycle of HP 4% v/v and surfactants were the samples proved to be negative. 

DU disconnected from the water supply and fed only with sterile water, as with the DU in the operating room of the HDC, are less contaminated and safer to use in invasive dental practices.

In conclusion, we assert that filter installation and shock disinfection with a solution of 4% v/v hydrogen peroxide and surfactants appear to be a promising alternative for decreasing *Legionella* colonization in DUWLs, although further field studies in other healthcare and community settings are required to confirm its effectiveness and its long-term efficacy in reducing biofilm. 

## 4. Materials and Methods

### 4.1. Setting

A 24-month investigation was carried out in a Hospital Odontostomatology Clinics (HOC) and in three Private Dental Clinics (PDC) housing 13 and 6 dental units, respectively. 

A validated checklist was used to identify the risk factors associated with contamination of DUWLs. The checklist included all the necessary items to create a schematic diagram showing the layout of the building’s water systems and DUWLs, as well as the information related to the compliance with good practices in controlling *Legionella* colonization.

The DUs examined here varied in model and year of installation: in the HOC, 8 Puma ELI, Castellani S.p.A., were installed in 2010 and 5 Skema 4, Castellani S.p.A. were installed in 2005; in the PDCs 2 C4+, Sirona Dental Systems GmbH, were installed in 2014, 2 LINEA 90, World Health Organization (WHO) OMS, were installed in 2007; and, finally, 2 Puma EVO 5, Castellani S.p.A., were installed in 2010. 

All devices were supplied through an independent system that received water from a 1-liter polypropylene bottle containing sterile water, manually filled. During the dental care activity, the municipal water fed the DUWLs by activating a switch when the bottle was running out of water. Contrastingly, the DU in the operating room was completely disconnected from the municipal water supply. In the hospital, the drinking water was softened and treated with a secondary chlorine dioxide disinfection (0.2–0.3 mg/L), while in private dental clinics the water was only softened.

In almost all DUs (Castellani and Sirona Dental Systems GmbH), at the end of the day, an automatic disinfection cycle was activated. According to the manufacturer’s recommendations, a disinfectant product that generated peracetic acid (0.2%) was applied. Only in one PDC, housing 2 OMS dental units, was disinfection performed manually on a daily basis through the application of 2.5% quaternary ammonium compounds. In the hospital, a procedure was written and shared with the dental clinic staff that was trained for the correct adoption of the DU management procedure. According to the procedure, all DUWLs were flushed for at least two minutes at the beginning and end of the day and after any significant period of inactivity. In addition, flushing was performed for at least 20–30 s between patients. No private clinic had a procedure where the DUs’ maintenance protocol was reported.

Following the results obtained from the microbiological investigations, a control program was started with more restrictive measures on the management of the quality of the supplied water, the periodic maintenance of the dental units (for example, the lubrication of the quick coupling seals on the handpiece, self-contained water bottles disinfection, etc.), the compliance with the flushing frequency, and the introduction of a shock disinfection procedure for the DUWL, where necessary.

A shock disinfection treatment was applied under the recommendations of the different dental unit manufacturers, when pathogens were isolated from the DUWL (the presence of *P. aeruginosa, Legionella* spp. or other pathogens). In particular, treatments were applied in all DUs except for the one of the operating room, which was disconnected from the water supply and fed only with sterile water.

### 4.2. Samplings and Microbiological Tests

Before the disinfection treatment, every three months, for each DU, 1.5 L of water was collected from the inlet, spittoon and handpiece. To assess the effectiveness of the treatment, sampling was carried out after 30 days. Moreover, 1.5 L of tap water was collected from a sink located near the DU. All water samples were collected without flushing before the start of professional activity.

The total microbial count at 22 and 37 °C, *Legionella* spp., *P. aeruginosa* and coliform bacteria were determined in all water samples, in accordance to the Italian and French guidelines [20,21]. One more liter of water was collected from the same devices for the detection of free-living protozoa (FLA) according to international standards [42].

Water temperatures were tested in all samples; residual chlorine concentrations were determined only in water sampled from taps and dental units fed from the municipal water network. 

*Legionella* spp. was detected as described by ISO11731:2017 [43]. One-liter samples were filtrated through a 0.2-µm membrane (Millipore, Billerica, MA, USA). The membrane was immersed in 10 mL of water and sonicated for 5 min to allow the bacterial cells to separate from the membrane. The resulting suspension was divided into three aliquots, the first one was brought to 50 °C to select *Legionella* spp. over the other bacterial species non heat resistant, the second one was added with 9 volumes of HCl-KCl acid solution, and the last one was plated with no treatment. A total of 0.1 mL of each aliquot was plated on Buffered Charcoal Yeast Extract (BCYE) Agar and Glycine Vancomycin Polymyxin Cycloheximide (GVPC) agar plates (Oxoid, UK), and incubated at 37 °C for 7–10 days in jars under an atmosphere containing 2.5% CO_2_. The suspect *Legionella* colonies were tested for species and serogroup by polyvalent agglutination latex test (Legionella latex test—Oxoid, UK).

The total microbial count was performed by inclusion in Plate Count Agar (Oxoid, UK) according to ISO 6222:1999 [44]. Coliform bacteria and *Pseudomonas aeruginosa* detection was performed by filtrating 100 mL of water through 0.45-µm membranes (Nalgene, Rochester, NY, USA). The membranes were laid on Endo Agar Les plates (Liofilchem, Italy) and Cetrimide Agar (Oxoid, UK) according to ISO 9308-1:2014 [45] for coliforms growth and ISO 16266:2006 [46] for *Pseudomonas aeruginosa* growth. The species’ confirmation of suspect colonies was obtained by Mini API galleries (bioMeriéux, Marcy-l’Étoile, France).

One-liter samples were filtrated through a 0.2-µm pore size of membrane (Millipore, Billerica MA, USA) for free-living protozoa search. The membrane filters were minced in 10 mL of sterile phosphate-buffered saline pH 7.2 (PBS), homogenized by vortex for 5 min and centrifuged at 1200 *g* for 15 min. A total of 200 μL of pellet was inoculated on Non-Nutrient Agar (NNA) with a lawn of heat-inactivated *Escherichia coli* in Page’s Amoeba Saline solution (PAS) and incubated at 37 °C. The presence of FLA was investigated by examining the NNA culture plates by inverted microscope Dmi1 (Leica Wetzlar, Germany) using 20× and 40× objectives.

From all positive samples, the growing amoebae were harvested from culture plates, placed in Eppendorf tubes and washed two times with PBS, pH 7.4, before molecular procedure. DNA extraction was performed by using the QIAamp DNA Micro Kit (Qiagen, Milan, Italy). To identify FLA species, the 18S rDNA amplification with primers P-FLA-F and P-FLA-R was performed, according to the protocol published by Cristina ML et al. [18]. 

### 4.3. Shock Disinfection Treatment

Shock disinfection treatments were applied in DUs housed in PDC and HOC, when contaminated by pathogens. Subsequently, the treatment was carried out every three months until an acceptable result was obtained. The effectiveness of disinfection was assessed after one month and in the subsequent sampling, scheduled every three months.

In PDC, dental units the disinfection was performed with a galenic formulation of 3% v/v hydrogen peroxide applied in dental units’ system for 1 h and followed by water flushing. 

In HOC, a shock disinfection was performed by using a galenic formulation of 3% and 6% v/v hydrogen peroxide and a second treatment was applied with a solution containing 4% v/v hydrogen peroxide and surfactants (Green Line Hydrogen Peroxide Cleaner 4%, Corcraft Product, Comstock, NY, USA). 

All disinfection treatments were carried out in a similar way, by using a biofilm removing system and a dye tracing to detect the disinfectant passage. The method of use was the following:

- Remove all hands and angle pieces to prevent blockages;
- Attach the provided connection adapter to the disinfectant bottle;
- Connect the device to the water inlet of the dental unit;
- Switch on the device and open the inlet valve to pump the disinfectant into the dental unit;
- As soon as purple liquid exits from the water-consuming units, switch off the device, close the valve and remove the device;
- Depending on the level of contamination of the dental unit, the action time may range between half an hour up to max 1 h;
- Upon completing the action time, turn on all water-consuming units until clean water runs out.

Membrane filters, 0.2 µm in size (Pall Corporation, New York, NY, USA), were set at the inlet of each dental unit to ensure a good microbial quality of the entrance water. The filters’ installation was done simultaneously to the first disinfection of the dental unit waterline in the PDC where they were installed after the treatment with 6% v/v hydrogen peroxide in HOC.

## Figures and Tables

**Table 1:** Results of the microbiological analysis performed on water samples collected from dental units housed in a Private Dental Clinic and in the Hospital Dental Clinic before shock disinfection.

		TMC 22 °C (Log CFU/mL)	TMC 37 °C (Log CFU/mL)	*P. aeruginosa* (P/A)	*E. coli* (P/A)	*Legionella* spp. (Log CFU/L)	*B. vesicularis* (P/A)	Free Living Protozoa (P/A)
**PDC**	Inlet	2.23 ± 0.30	2.06 ± 0.05	A	A	0	P	A
	Spittoon	2.43 ± 0.40	2.16 ± 0.07	P	A	4.06 ± 0.09	A	A
	Handpiece	2.52 ± 0.42	2.49 ± 0.19	A	A	4.24 ± 0.12	A	A
	% of positive DUs	100% (6/6)	100% (6/6)	33% (2/6)	0% (0/6)	33% (2/6)	33% (2/6)	0% (0/6)
**HDC**	Inlet	3.14 ± 0.12	3.35 ± 0.23	A	A	3.54 ± 0.21	A	P
	Spittoon	3.25 ± 0.19	3.53 ± 0.32	P	A	4.30 ± 0.17	A	A
	Handpiece	3.42 ± 0.20	3.66 ± 0.27	P	A	4.59 ± 0.07	A	A
	% of positive DUs	85% (11/13)	85% (11/13)	85% (11/13)	0% (0/13)	31% (4/13)	0% (0/13)	46% (6/13)

Note = TMC: Total Microbial Count; P/A: Presence or Absence; PDC: Private Dental Clinic; HDC: Hospital Dental Clinic.

**Table 2:** Results of the microbiological analysis performed on water samples collected from dental units housed in a Private Dental Clinic and in the Hospital Dental Clinic after shock disinfection.

		TMC 22 °C (Log CFU/mL)	TMC 37 °C (Log CFU/mL)	*P. aeruginosa* (P/A)	*E. coli* (P/A)	*Legionella* spp. (Log CFU/L)	*B. vesicularis* (P/A)	Free Living PROTOZOA (P/A)
**PDC** **(HP 3%)**	Inlet	1.60 ± 0.05	1.66 ± 0.36	A	A	A	A	A
	Spittoon	1.72 ± 0.17	1.87 ± 0.02	A	A	A	A	A
	Handpiece	2.03 ± 0.05	1.93 ± 0.04	A	A	A	A	A
	% of positive DUs	100% (2/2)	100% (2/2)	0% (0/2)	0% (0/2)	0% (0/2)	0% (0/2)	0% (0/2)
**HDC** ** (HP 3%)**	Inlet	2.53 ± 0.41	2.25 ± 0.31	A	A	1.18 ± 0.64	A	A
	Spittoon	2.62 ± 0.40	2.62 ± 0.25	P	A	1.79 ± 0.32	A	A
	Handpiece	2.74 ± 0.30	3.41 ± 0.58	P	A	2.47 ± 0.41	A	A
	% of positive DUs	67% (8/12)	92% (11/12)	83% (10/12)	0% (0/12)	8% (1/12)	0% (0/12)	0% (0/12)
**HDC** ** (HP 6%)**	Inlet	1.50 ± 0.43	2.20 ± 0.05	A	A	0	A	A
	Spittoon	1.69 ± 0.21	2.56 ± 0.23	P	A	0	A	A
	Handpiece	1.96 ± 0.03	2.84 ± 0.03	P	A	0	A	A
	% of positive DUs	90% (9/10)	100% (10/10)	100% (10/10)	0% (0/10)	0% (0/10)	0% (0/10)	0% (0/10)
**HDC (HP 4%** **and surfactants)**	Inlet	0	0	A	A	0	A	A
	Spittoon	0	0	A	A	0	A	A
	Handpiece	0	0	A	A	0	A	A
	% of positive DUs	0% (0/10)	0% (0/10)	0% (0/10)	0% (0/10)	0% (0/10)	0% (0/10)	0% (0/10)

Note = TMC: Total Microbial Count; P/A: Presence or Absence; HP: hydrogen peroxide; PDC: Private Dental Clinic; HDC: Hospital Dental Clinic.
